# Professionalism skills education in medical physics residency: Current state and perceived importance

**DOI:** 10.1002/acm2.70096

**Published:** 2025-04-24

**Authors:** Anna Rodrigues, Leah Schubert, Laura Padilla, Kristi Hendrickson, Hania Al‐Hallaq, Irina Vergalasova

**Affiliations:** ^1^ Duke University Medical Center Durham North Carolina USA; ^2^ University of Colorado School of Medicine Aurora Colorado USA; ^3^ University of California, San Diego La Jolla California USA; ^4^ University of Washington Seattle Washington USA; ^5^ Emory University Atlanta Georgia USA; ^6^ Rutgers Cancer Institute of New Jersey New Brunswick New Jersey USA

**Keywords:** professionalism, residency education, soft skills training

## Abstract

**Purpose:**

The purpose of this study was to collect data on current practices for teaching and assessing professionalism in CAMPEP‐accredited residency programs.

**Methods:**

A survey of 21 questions was sent to 160 program directors (PDs) of CAMPEP‐accredited residency programs. A list of professionalism skills was compiled from the AAPM MPLA curriculum: (a) Personal and interpersonal, (b) professional and developmental, and (c) executive and administrative. The survey collected information on: (1) residency program respondent demographics, (2) essential professionalism skills and training methods, (3) confidence and satisfaction in teaching professionalism, (4) assessment of professionalism, (5) barriers and desired resources, (6) training of PD and staff in professionalism, and (7) free response. Descriptive statistics and thematic analyses were used to evaluate the collected data.

**Results:**

A total of 97 respondents completed the survey (therapy = 75, diagnostic = 22) with a 61% response rate. 16 out of 24 professionalism skills were deemed essential for trainees to develop during residency training. While 92% teach professionalism, only 51% reported confidence in teaching these skills. The ABR/ACR/RSNA/AAPM/ASTRO/ARR/ARS online modules are used by 87% but only 31% indicated that the modules are sufficient. Only 10% use a structured method for assessment. The majority (59%) assess professionalism in an ad‐hoc manner and 22% only assess when problems arise. 44% reported facing barriers to implementing a professionalism curriculum. The main barriers for developing a professionalism curriculum included: lack of time (39%), resources (32%), or expertise (26%). 79% reported that case studies were the most desired resource. 47% of respondents indicated receiving formal professionalism training.

**Conclusions:**

There is a strong need and desire for structured professionalism training in residency programs. This study presents a consensus understanding of the professionalism skills that are deemed essential and teachable during residency and has identified areas for improvement in teaching, assessing, and developing curricula.

## 1 INTRODUCTION

Medical physics is an interdisciplinary field that requires graduate education followed by residency training to become eligible to take American Board of Radiology (ABR) certification exams necessary to work as a qualified medical physicist. Programs are accredited by The Commission on Accreditation of Medical Physics Education Programs (CAMPEP) which sets accreditation standards for didactic and clinical curricular requirements including professionalism and ethics.^1^ Some professionalism examples included in CAMPEP standard 8.6 are: the elements of professionalism, the do's and don'ts of professionalism, and how to judge professionalism. Aside from CAMPEP requirements, the American Association for Physicists in Medicine (AAPM) Report 249^2^ on the “Essentials and guidelines for clinical medical physics residency training programs” provides recommendations and guidance on curricular content including professionalism and ethics training. Published in 2013, the report states that “residents shall develop professionalism by: demonstrating a commitment to carrying out responsibilities; understanding professional issues and participating in the activities of professional societies; showing leadership and adhering to ethical principles; demonstrating sensitivity to diverse patient populations; being responsive to the needs of patients and prioritizing those needs over self‐interest; respecting patient privacy and confidentiality; and demonstrating a commitment to excellence and ongoing professional development.” Further, one of the five ABR categories on the exams to achieve board certification includes professionalism and ethics.^3^

Professionalism is a critical aspect of working in an allied‐health field such as medical physics. Professionalism encompasses a variety of different behaviors, mindsets, and skills, which should be well‐defined and integrated into medical physics residency training. To date, there has not been an explicit listing of the specific professionalism skills that should be taught during residency training. Neither CAMPEP standards nor TG‐249 recommendations provide a detailed list of professionalism skills to be learned by the end of residency. In contrast, AAPM TG‐159 report provides such a list for ethics subjects.^4^ A commonly accessed resource for professionalism and ethics training for medical physics residents is the online modules, which is co‐sponsored by multiple professional societies including AAPM.^5^ Of the 11 modules, only four are dedicated to professionalism.

There have been recent developments to better define professionalism skills for medical physicists in the context of leadership. The AAPM Medical Physics Leadership Academy (MPLA) has developed a list of professionalism skills deemed important to practicing medical physicists including personal and interpersonal, professional and developmental, and executive and administrative skills.^6^ It is currently unclear which of these professionalism skills should be incorporated into residency training, yet they are recommended by TG‐249, required by CAMPEP, and residents are evaluated on them during ABR examinations.

Furthermore, there is a lack of guidance on how to teach and assess professionalism in a residency program. While there is an expectation that medical physics residency PDs and associated training staff teach and assess professionalism, it is likely that most of the PDs have never received such training themselves. Ozturk et al. surveyed AAPM membership and found that 60% of respondents did not receive professionalism and ethics education during their medical physics training.^7^ Since 2013, there has been no further investigation or follow‐up on the appropriate curriculum for professionalism training for medical physics residents, much less on how it should be taught or evaluated.

These challenges are not unique to medical physics residency training programs: For physicians, professionalism is also a key aspect of residency training, as it is one of the six core competencies specified for residency programs accredited by the Accreditation Council for Graduate Medical Education (ACGME).^8^ Professionalism in medicine was codified by the ACGME in 2007 requiring documentation of this education and evaluation during residency training. Since this time, the teaching and assessment of professionalism during medical residency training has been the subject of medical education research as it was noted to be one of the most difficult topics to teach and assess while also being a fundamentally vital skill that ultimately impacts patient safety.^9^, ^10^, ^11^

The goals of this survey were to (1) understand the importance of specific professionalism skills for residency programs based on the MPLA curriculum, (2) collect and present the data on current practices of teaching and assessing professionalism in CAMPEP‐accredited residency programs, and (3) identify barriers and resources to support program directors (PDs) in formalizing professionalism training.

## 2 METHODS

A working group of educators interested in professionalism training and serving on educational committees, including the AAPM Medical Physics Residency Training and Promotion Subcommittee (MPRTP), the SDAMPP Professional Issues Committee, and the SDAMPP Education Practices Committee was formed to work on this initiative. A short pilot survey was initially sent out to 15 PDs to assess the need, which prompted the working group to design a comprehensive survey via a consensus‐based approach and using methodologies for questionnaire design commonly used in survey research. A survey of 21 questions divided into seven sections was developed to collect information from PDs of CAMPEP‐accredited residency programs in the US and Canada. The eight sections were:

1. Residency program respondent demographics
2. Essential professionalism skills
3. Professionalism training methods
4. Assessment of professionalism
5. Confidence and satisfaction in teaching professionalism
6. Barriers and desired resources
7. Training of PDs and staff in professionalism
8. Free response

To define essential professionalism skills, PDs were asked about 24 specific skills grouped into three categories adapted from the MPLA curriculum for medical physicists^6^ for consistency and to maintain a level of standardization within medical physics: (a) personal and interpersonal skills, (b) professional and developmental skills, and (c) executive and administrative skills. The full survey instrument can be found in the supplemental data.

The survey was further refined by first performing a cognitive interview with an independent reviewer, which involved verbal discussion and evaluation of question intent and interpretation.^12^ Upon incorporating feedback from the qualitative evaluation, the survey then underwent quantitative evaluation by an independent group of content experts. For the purpose of this work, content experts were defined as medical physicists directly involved in residency program education and/or administration. Six content experts evaluated each survey question and response option using a form adapted from Gehlbach et al.^13^ and Rubio et al.^14^ They rated the clarity and relevance of each question using a 1–4 scale. The ratings were then analyzed to determine: the reliability agreement (RA), which is the extent to which the experts agree on their ratings; and the content validity index (CVI), which quantifies the collective level of the experts’ validity ratings. The RA was calculated by counting whether all experts rated an item for clarity and relevance in one of two groups: Unclear or irrelevant (questions rated as 1 or 2) and clear or relevant (rated as 3 or 4). There was 90% agreement for relevance, and 85% for clarity amongst the six content experts. Regarding CVI, the experts agreed that 98% of the questions were relevant and 97% were clear. Questions and response options with a CVI < 1.0 were then removed from the survey of revised accordingly. The full results can be found in the Supplemental Data.

The study was submitted for IRB review and deemed exempt. The finalized survey was distributed in May 2023 via a REDCap^15^ electronic data capture hosted at the University of Washington and supported under grant UL1 TR002319. The survey was sent to a total of 160 residency PDs (121 therapy and 39 diagnostics) listed on the CAMPEP website.^16^ The survey was open for 3 months. Three reminder emails were sent.

The results from the survey were analyzed using descriptive statistics. A thematic analysis was performed by the five authors for the text response question of the survey: “Please share if there is anything else you wish to add about professionalism training.” The analysis was done in two phases. In the first phase, each author independently identified keywords or excerpts from individual responses. In the second phase, the pattern of codes was reviewed to establish overall themes.

## 3 RESULTS

### 3.1 Residency program respondent demographics

Of 115 responses, 97 complete responses (75 from therapeutic and 22 from diagnostic residency programs) were obtained and subsequently included in the analysis for a response rate of 61%. All respondent demographics are shown in Table 1.

**TABLE 1:** Residency program respondent demographics.

Survey response (*n* = 160)	
Total responses	72% (115)
Complete response	61% (97)
Incomplete response	4% (7)
No response	7% (11)
**Respondent's role (*n* = 97)**	
Program director	95% (92)
Associate program director	3% (3)
Other	2% (2)
**Affiliation (*n* = 97)**	
Affiliated with a university	68% (66)
Not Affiliated with a university	30% (29)
Other	2% (2)
Affiliated with a graduate program	36% (35)
Not affiliated with a graduate program	64% (62)
**Physics specialization (*n* = 97)**	
Therapeutic	77% (75)
Diagnostic	12% (12)
Diagnostic + nuclear medicine	10% (10)
**Total # of residents (*n* = 97)**	
0	2% (2)
1	13% (13)
2	38% (37)
3	11% (11)
4	19% (18)
5–10	14% (14)
>10	2% (2)
**Length of training period (*n* = 97)**	
2 years	81% (79)
> 2 years	19% (18)

Most respondents were from residency programs affiliated with a university (68%), but only a minority were affiliated with a medical physics graduate program (36%). Respondents indicated specializations in therapeutic (77%), diagnostic (12%), and diagnostic + nuclear medicine (10%) physics. These demographics are representative of the overall distribution of specialization from all CAMPEP‐accredited programs: therapeutic (76%), diagnostic (17%), and diagnostic + nuclear medicine (7%) residency programs.^17^ Most respondents (81%) indicated a training duration of 2 years. Program sizes represented in this survey were evenly split between small programs with ≤2 residents (54%) and large programs ≥3 residents (46%).

### 3.2 Essential professionalism skills

**FIGURE 1:**
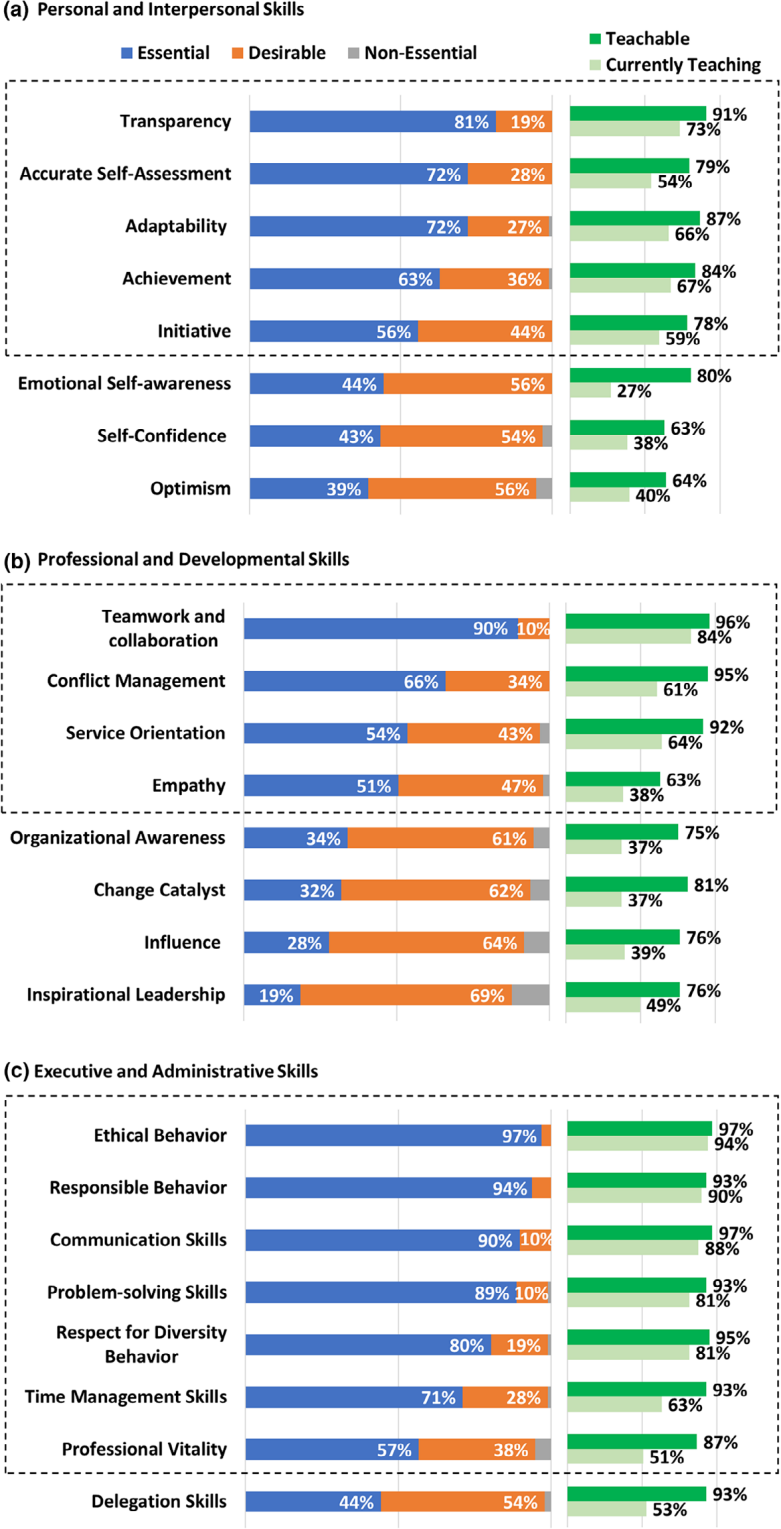
PDs were asked to rate specific professionalism skills grouped into categories: (a) Personal and interpersonal skills, (b) Professional and developmental skills, and (c) Executive and administrative skills. Respondents indicated how essential and teachable they deemed each skill in addition to whether they were teaching it. The dashed box indicates the skills deemed essential by the majority of respondents.

Most respondents (92%) reported teaching professionalism to their residents. Figure 1 shows the specific professionalism skills as well as whether PDs deemed them to be essential or teachable, and whether programs were currently teaching the skill. Sixteen out of the 24 skills were deemed essential by most (>50%) of the respondents Table 2. The remaining eight skills (emotional self‐awareness, self‐confidence, optimism, organizational awareness, change catalyst, influence, inspirational leadership, and delegation skills) were still deemed desirable for residents to develop during their training.

**TABLE 2:** List of 16 professionalism skills deemed essential for residents to develop during training by the majority of respondents.

Personal and interpersonal skills	Professional and developmental skills	Executive and administrative skills
Transparency Accurate self‐assessment Adaptability Achievement Initiative	Teamwork and collaboration Conflict management Service orientation Empathy	Ethical behavior Responsible behavior Communication skills Problem‐solving skills Respect for diversity in behavior Time management skills Professional vitality

It is important to emphasize that all 24 skills were deemed teachable by most (>50%) respondents. The percentage of respondents who deemed skills teachable was always higher than the percentage of respondents currently teaching the skill. There are wide variations in the gap between a teachable skill and whether it is being taught. This gap is small for some skills, such as ethical behavior (3%), but large for other skills, such as emotional self‐awareness (53%).

**FIGURE 2:**
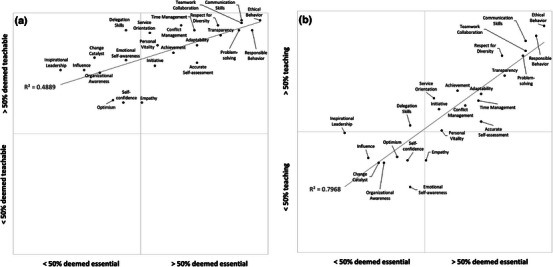
An Eisenhower‐type scatter plot of all 24 professional skills comparing the percentage of respondents who indicated that the skill was essential for residents to develop during training, whether it is a) teachable and b) currently being taught.

Figure 2 shows a scatter plot of all 24 professionalism skills comparing how essential skills were deemed versus whether the skill was teachable (Figure 2a) or being taught (Figure 2b). The skills/behaviors that were deemed essential were highly correlated with whether the skill was currently being taught (*R* ^2 ^= 0.79) but less correlated with whether the skill was teachable (*R* ^2^ = 0.49). The upper‐right quadrant of the plot indicates skills that are considered essential and teachable or are being taught by the majority of respondents. The lower quadrants indicate skills that are not being taught or are not considered teachable by the majority of respondents even when they are considered by the majority to be essential (lower‐right) or the minority to be essential (lower‐left). Skills in the lower‐right quadrant may indicate a gap in training. One skill that fell into that quadrant was empathy. It was deemed essential by 51% of respondents and also cited as teachable by 63% of respondents, but only 37% stated they were currently teaching it. The upper‐left quadrant indicates skills that are being taught, but not considered essential, however they were deemed highly teachable.

### 3.3 Professionalism training methods

**FIGURE 3:**
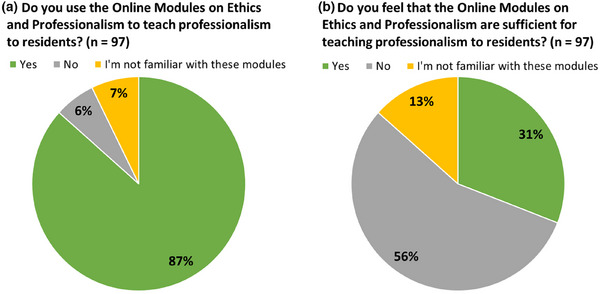
Percentage of respondents indicating whether or not (a) they currently use the online modules on ethics and professionalism to teach professionalism to residents and whether or not, (b) they feel that the modules are sufficient for teaching professionalism.

Regarding how professionalism is being taught, PDs were asked about the Online Modules on Ethics and Professionalism.^5^ The majority (87%) of respondents use the modules in residency training. Despite the prevalent use of online modules, only 31% of respondents felt that the modules were sufficient, while 56% felt that they were not sufficient as shown in Figure 3. There is a larger percentage of respondents who indicated a lack of familiarity with the online modules to determine whether they are sufficient for teaching Figure 3b. One possible explanation for this result is, while respondents may be aware of the online modules to use them in their program, not all respondents may be familiar enough with the modules to determine their effectiveness.

**FIGURE 4:**
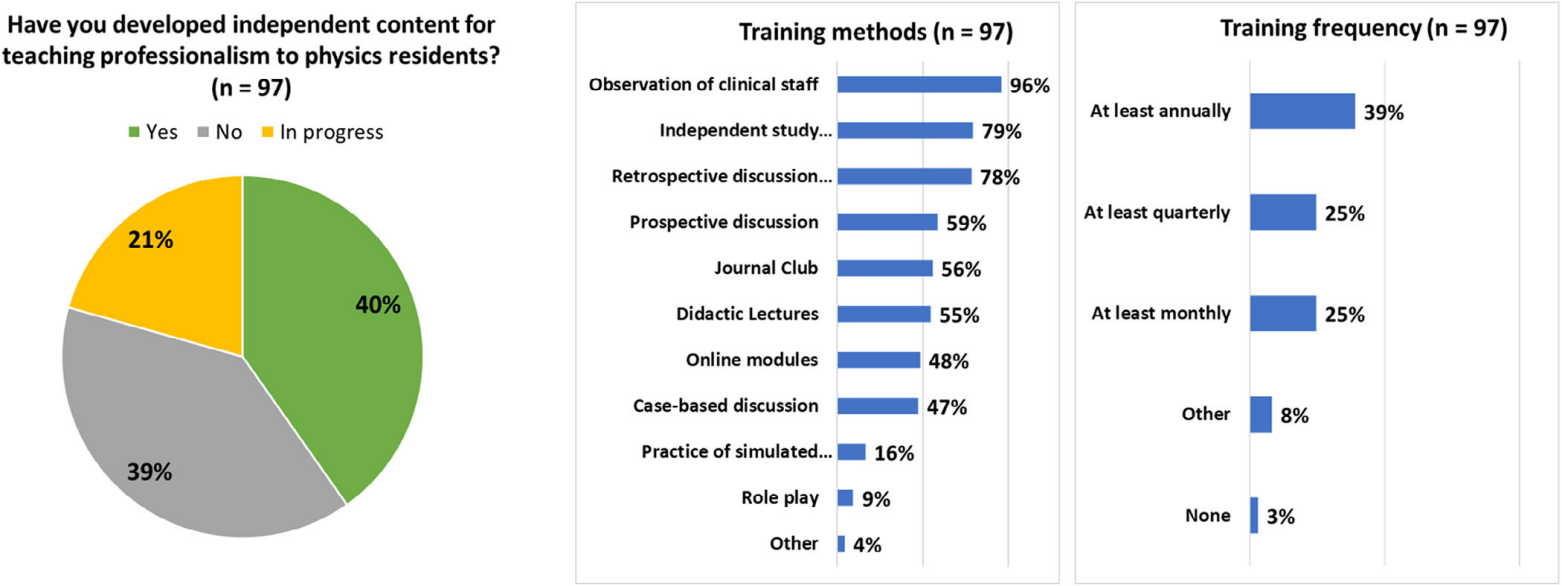
Training methods and training frequency as reported by respondents (*n* = 97).

PDs were asked if they have developed independent content for teaching professionalism to physics residents. While 39% have not developed their own resources, 40% stated that they have developed resources and 21% are currently developing resources. Free text responses describing these resources ranged from adaptation of existing materials (e.g., institutional new employee training modules, biomedical research online training modules, AAPM MPLA materials, AAPM Virtual Library presentation, online videos, outside experts, reading assignments) to the development of new resources (e.g., courses, lectures, oral exam style questions, workshops, seminars, group activities and assignments, discussions, panel colloquia, case studies, and active learning techniques). The most common method of teaching professionalism was observation of clinical staff (used by 96% of respondents), and >50% used independent study and/or self‐reflection, retrospective discussion in response to an event, prospective discussion, journal club, or didactic lectures Figure 4. Explicit professionalism training is provided at least annually (39%), quarterly (25%), or monthly (25%) as shown in Figure 4.

### 3.4 Assessment of professionalism

Regarding the assessment of residents’ professionalism, only 10% of respondents use a structured assessment method. Otherwise, professionalism is assessed on an ad‐hoc basis (59%), only when problems/issues arise (22%), or not at all (9%). Of the respondents who assess professionalism in some fashion (*n* = 88), the most common assessment methods were direct observation by rotation supervisors or equivalent (94%), followed by indirect observation reported by others (66%), and professionalism‐specific questions during an exam (35%).

**FIGURE 5:**
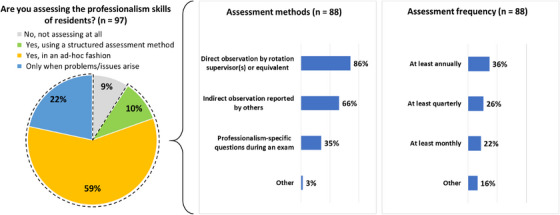
Assessment methods and frequency reported by respondents (*n* = 97).

Assessment of professionalism is performed at least annually (36%), quarterly (26%), monthly (22%), or at another frequency (16%) including: continuous, per rotation, or once per rotation. These results are summarized in Figure 5. Feedback is provided to the resident regarding professionalism in many ways. Overwhelmingly, verbal discussion with the resident (90%) was the top feedback mechanism, while a minority of respondents stated that they provided written feedback either with a score (12%) or comments (33%).

### 3.5 Confidence and satisfaction

**FIGURE 6:**
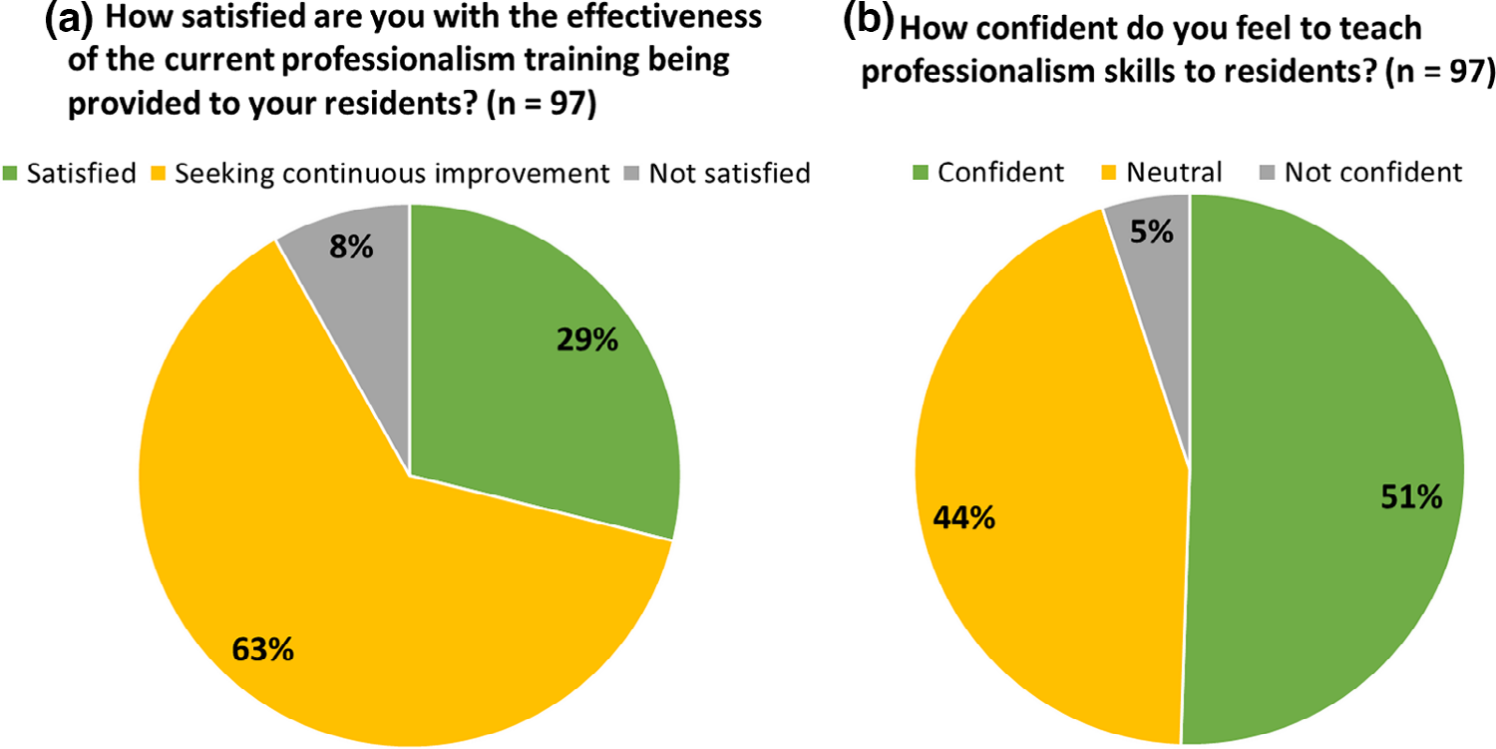
Percentage of respondents indicating their (a) satisfaction with the effectiveness of their current professionalism training and (b) confidence in teaching professionalism to residents.

While 92% of respondents teach professionalism, only 51% indicated confidence to teach this content, while 44% were neutral and 5% were not confident. Furthermore, 69% indicated they are continuously seeking improvement of their curriculum and 29% indicated being satisfied with the effectiveness of their current training. Only 8% indicated dissatisfaction with the effectiveness of their current curriculum. These results are summarized in Figure 6.

### 3.6 Barriers and desired resources

PDs were asked whether they faced barriers to teaching professionalism with 44% indicating “yes”. Respondents reported a lack of time (88%), resources (72%), expertise (58%), and/or interest (14%). Five percent cited other reasons, including a lack of time for both faculty and trainees and the inability to assess teaching effectiveness.

**FIGURE 7:**
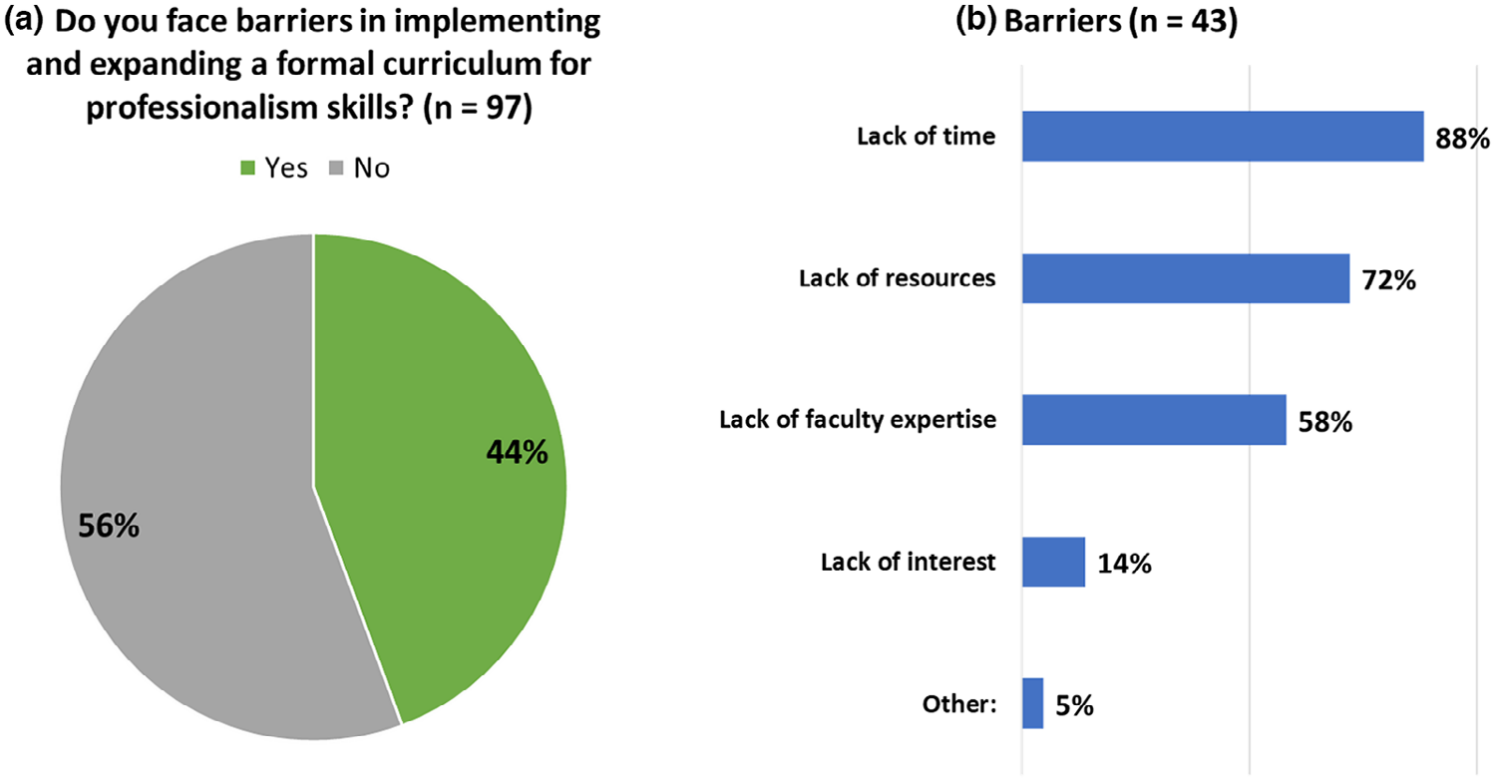
Percentage of respondents (*n* = 97) who (a) face barriers in implementing and expanding a formal curriculum for professionalism. (b) 43 out of 97 respondents (44%) indicated what barriers they experience to teaching professionalism in residency.

**FIGURE 8:**
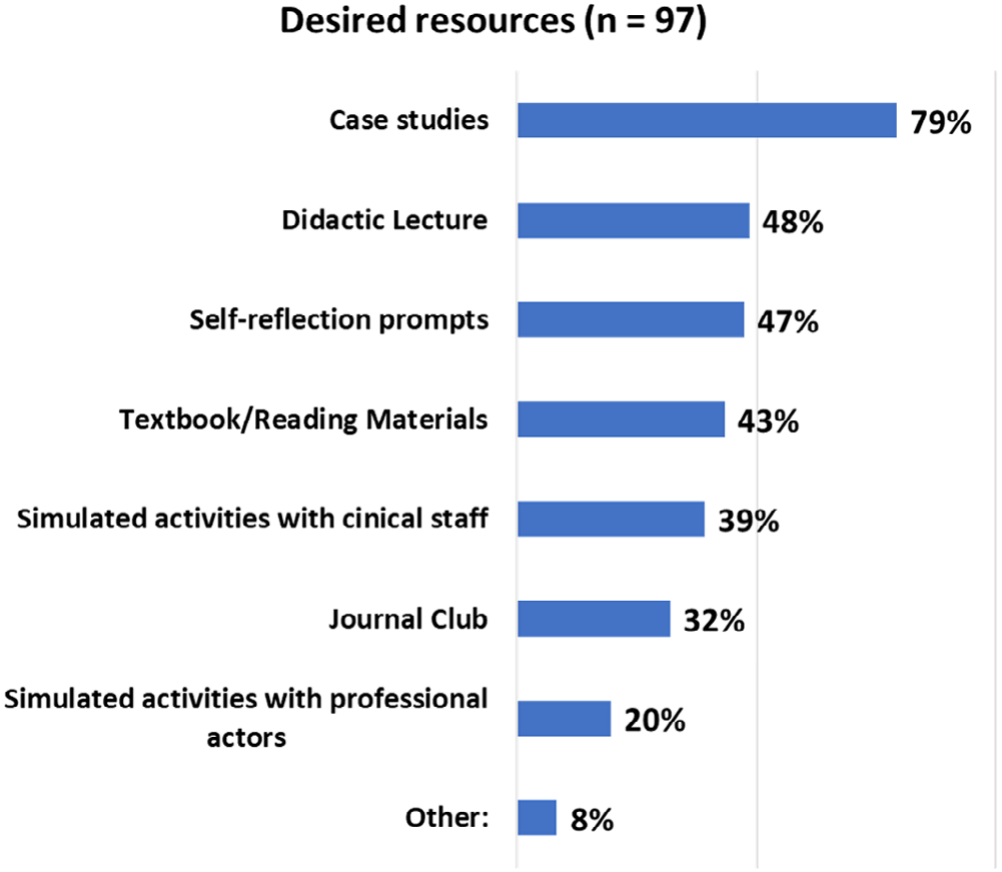
Resources desired by residency programs for teaching professionalism (*n* = 97).

Case studies were the most desired resource for teaching professionalism. In order of preference, other desired resources were didactic lectures, self‐reflection prompts, and textbook/reading materials. These results are shown in Figures 7 and 8.

### 3.7 Training of program director and program faculty in professionalism

When asked about their personal history of formal training in professionalism (e.g., didactic courses, workshops) 47% of respondents reported receiving formal training while 53% had not. For the respondents who received formal training, the majority (63%) felt that receiving training in professionalism was important in order to teach it, while only 43% of the respondents who did not receive formal training felt the same Table 3. A chi‐square test of independence showed that there was no significant association between respondents that had received formal training and their perception of its importance for teaching professionalism (Yates chi‐square *p* = 0.09).

**TABLE 3:** Contingency table of the perceived importance of receiving professionalism training separated by respondents who themselves had received formal training or not.

	Important	Neutral	Not important
**Formal training (*n* = 46)**	63% (29)	28% (13)	9% (4)
**No formal training (*n* = 51)**	43% (22)	53% (27)	4% (2)

### 3.8 Free response

Of 97 respondents, 20 provided written comments about professionalism training. The three overall themes were: the need for shared resources (*n* = 10), the challenges in selecting appropriate teaching and assessment methodologies (*n* = 5), time constraints during training (*n* = 5), and the challenge of the hidden curriculum (*n* = 4). Selected examples of these responses are shown in Table 4.

**TABLE 4:** Select free text responses demonstrating each of the four common themes.

Common themes	Example responses
**Need for shared resources**	“Development of shared resources in any form would be very helpful to many programs.”
**Challenges in teaching and assessment**	“Extremely broad topic and think it is difficult to assess the effectiveness of the training.”
**Time constraints**	“All the skills are very useful and some are essential. But residency is only 2 years and very busy with clinical tasks and trainings. We can only focus on the skills that are directly related to clinical service and training (e.g., ethics, communication).”
**Hidden curriculum**	“Challenging timewise to devote a lot of time to explicit teaching, so often a part of the hidden curriculum.”

## 4 DISCUSSION

### 4.1 Perceived importance and current teaching of specific professionalism skills

While professionalism training is required in medical physics residencies. There is no consensus on which professionalism skills are most relevant to medical physics. When asked about 24 professionalism skills from the MPLA curriculum, respondents indicated that 16 of 24 skills are essential for residents to develop during training, while the remaining 8 skills were considered desirable (but not essential). Skills not deemed essential by PDs fell into two categories. The first are those that might be perceived to be personal attributes rather than directly measurable (i.e., emotional self‐awareness, self‐confidence, optimism). The second included skills related to leading others (e.g., organizational awareness, influence, delegation), which may not be the focus during residency. The list of professionalism skills identified by PDs to be essential could be used to help guide the development of residency training resources.

The largest difference was for empathy, which was considered essential by 51% of respondents but is only taught in 38% of programs. Research has shown that empathy is a skill that can be developed and is important in workplace leadership and for healthcare workers.^16^, ^18^ This finding is notable, considering wellness and professional burnout has received increasing attention. A recent study of resilience and wellbeing in medical physics residency programs revealed the demanding nature of residency programs, highlighting various sources of burnout, and strategies for support and interventions.^19^ Additional attention to developing ways to teach empathy and professional vitality in residency programs may be warranted.

The correlation between whether skills are being taught and whether they are considered essential was stronger than whether they are considered teachable. The data, however, only demonstrates correlation and does not provide information about causation.

### 4.2 Current methods of teaching professionalism

The AAPM online modules are used by most programs (87% of respondents). While these modules provide a centralized resource available to all AAPM members that is widely used by residency programs, the majority of respondents did not indicate that these online modules were sufficient for teaching professionalism to residents. Thus, programs have developed or are developing their own content for teaching professionalism, with the three most common training methods being observation of staff, independent study, and retrospective discussion in response to an event, possibly because these activities are relatively easy to implement. It should be noted that the three least common methods (case‐based discussion, practice of simulated situations, and role play) are evidence‐based teaching methods that are effective in medical education but may take more time and effort to develop.^20^, ^21^, ^22^ There appears to be a mismatch between the content currently being developed and these evidenced‐based effective teaching strategies. Note that this study only collected data on how professionalism, as an overall topic, was being taught. It is possible that various professionalism skills are more suitable to certain types of pedagogical methods.

### 4.3 Current methods of assessing professionalism in residents

This study identified a gap in the formal assessment of professionalism during residency. Only 10% of respondents assess professionalism in a structured way. The majority assess professionalism in an ad hoc fashion (59%), while a large percentage (31%) assess only when problems arise or not at all. Furthermore, feedback is given to the resident primarily through verbal means (90%) while only 12% provide structured written feedback with a score and 33% provide written comments.

This finding could be due to the following factors: lack of specific guidance, difficulty in assessing professionalism in general, and the use of teaching strategies tied to the assessment strategies. For example, it may be easier to create an objective assessment method when using a controlled, pre‐defined role play or case‐based teaching strategy, as opposed to teaching professionalism with observations only. In medical residencies, the ACGME lists professionalism in their six core competencies, and therefore requires ACGME‐accredited programs to assess professionalism explicitly. While CAMPEP requires ethics and professionalism to be taught in a CAMPEP‐accredited residency and also requires that programs evaluate their residents and have a process for how residents progress through the program, there is no requirement on the specific skills and behaviors that should be assessed. Assessment of professionalism is a challenging topic and has been the subject of medical education research.^10^, ^11^, ^12^ An assessment framework can be developed once the key professionalism skills are clearly defined. The results of our study may provide a starting point for clearly defining the scope of professionalism topics, teaching methodologies, and assessments for medical physics residency.

### 4.4 Satisfaction and confidence in teaching professionalism

This study has also identified a large need to address the effectiveness of professionalism training. Only 29% of respondents were satisfied with the effectiveness of the current professionalism training provided to their residents. Note that 63% of respondents indicated that they were seeking continuous improvement in their current professionalism training for residents. This result indicates a positive interest in improving the current state of professionalism training in residency programs. Additionally, approximately only half (51%) of respondents indicated confidence in teaching professionalism.

### 4.5 Barriers and desired resources

The barriers reported are similar to those found in medical and other allied healthcare training programs and include a lack of time, resources, and/or faculty expertise to implement a formal curriculum for professionalism training during residency.^23^, ^24^, ^25^ Overcoming these barriers effectively requires a multi‐faceted approach. Individual residency programs are time‐ and resource‐limited. Professionalism training in medical physics residency programs would benefit from a model of best practices and explicit consensus of the medical physics educator community on the specific professionalism skills to teach and how to teach and evaluate them. Shared resources could present solutions to address the resource limitations, by eliminating the need to individualize or reinvent the wheel for each program. The use of inter‐institutional expertise and resources from outside the residency program, such as the AAPM MPLA cohorts^26^ or the AAPM Multi‐Institutional Journal Clubs for Residency Programs,^27^ may be viable options to “outsource” both the time, expertise, and resources.

Case studies were found to be the most desired resource. AAPM's MPLA Cases Subcommittee and Ethics Case committee both have generated content and training formats (cohorts).^28^ Simulated activities were one of the least desired resources yet are one of the most effective methods for professionalism training and assessment as they can provide immediate feedback and reflection by the learner in a controlled environment, therefore allowing the learner to refine their skills without real‐world impacts.^29^

### 4.6 Professionalism training of program directors and perceived importance of training

This study found that PDs, who are expected to teach professionalism, had received limited formal training in professionalism themselves. Less than half of respondents (47%) had received professionalism training, a rate that is similar to that from a 2013 survey of AAPM members in which a minority (40%) reported having received professionalism and ethics education during their medical physics training.^7^ The low rate of PDs who have been formally educated in professionalism could be one potential reason for the low confidence and satisfaction reported in providing such training to their residents.

Respondents’ views are divided on the importance of receiving professionalism training in order to teach it, with approximately half indicating a positive correlation between their individual educational experience and their ability to teach professionalism. One limitation of this study is that further details about PD's formal training, such as the extent of training and teaching methods experienced, were not included in the survey questions.

### 4.7 Professionalism in the hidden versus stated curriculum

In educational literature, it is understood that trainees learn from the stated curriculum as well as the hidden curriculum.^30^ The hidden curriculum represents skills that are not included explicitly in the stated curriculum in addition to actions observed by the trainees that are accepted by others in the profession. If explicit curricula and assessment tools for professionalism are not required in medical physics, this may be perceived by trainees as minimizing its importance to our field. Additionally, if we do not explicitly teach professionalism in a deliberate manner, trainees will mainly learn from observing those around them and could potentially absorb unwanted professional skills. The multiple effects of the hidden curriculum on professionalism have been documented in the graduate medical education literature.^30^ As this survey has elucidated that many educators have not received professionalism training, it may be worthwhile for medical physics to initially utilize a professionalism curriculum that can include both trainees and educators (i.e., group‐based discussions). This would enlist current practitioners to continuously improve their professional skills while also emphasizing its importance to our field.

### 4.8 Limitations and future work

A common barrier identified in this study was the limitation of time within a residency program. The coordination of professionalism training between graduate and resident programs could be a way to mitigate this time limitation as well as to emphasize the importance of these skills as early as graduate school. The goal of this work was not to collect data on which training methods are effective for specific professionalism skills, nor to address how to assess these skills in a way that is meaningful to the resident and the program. While this study revealed a need for improved teaching and assessment of these skills, how best to fill those needs is the subject of future research.

Furthermore, this study only surveyed PDs. The perspective of current and recently graduated trainees on professionalism is also of value in developing adequate professionalism training for residency programs. It is important to remember that trainees may have a unique perspective on what they consider effective for their professionalism training. A future study should survey this population.

To the authors’ knowledge, this study is the first to systematically collect the data from medical physics residency PDs specifically on the current status of professionalism training in medical physics residency programs. It serves as a data‐driven foundation that identifies the need for generating professionalism modules for residency programs, which is the goal of future work. The results presented here can inform future recommendations that further define specific professionalism skills, and how best to teach and assess them, during residency. Pooling resources across programs and harnessing strategies that employ a team approach to professionalism education may be a strategy for reducing the barrier for generating professionalism curricula.

## 5 CONCLUSION

Professionalism skills are required in medical physics residency training by CAMPEP. A strong need and desire for structured professionalism training in residency programs was reported by the 97 PDs who responded to our survey. This study presents a current understanding of the professionalism skills that are deemed essential and teachable during residency. Furthermore, it identifies actionable areas in the teaching and assessment of these skills that we, as a field, can work on to decrease barriers for professionalism training during residency.

## AUTHOR CONTRIBUTIONS

The authors confirm contribution to the paper as follows: *Study conception and design*: Anna Rodrigues, Leah Schubert, Laura Padilla, Kristi Hendrickson, Hania Al‐Hallaq, Irina Vergalasova. *Data collection*: Irina Vergalasova.*Analysis and interpretation of results*: Anna Rodrigues, Leah Schubert, Laura Padilla, Kristi Hendrickson, Hania Al‐Hallaq, Irina Vergalasova. *Draft manuscript preparation*: Anna Rodrigues, Leah Schubert, Laura Padilla, Kristi Hendrickson, Hania Al‐Hallaq, Irina Vergalasova. All authors reviewed the results and approved the final version of the manuscript.

## CONFLICT OF INTEREST STATEMENT

The author declares no conflicts of interest.

## Supporting information



Supporting Information

Supporting Information
