# Supplementary material for: Professionalism skills education in medical physics residency: Current state and perceived importance
Source: J Appl Clin Med Phys. 2025 Apr 24;26(6):e70096. doi: 10.1002/acm2.70096 (PMC12148774; doi:10.1002/acm2.70096)
Supplement: Supplementary file 1 — Supporting Information [file ACM2-26-e70096-s002.pdf]

Professionalism Survey

This survey was developed to assess how professionalism is taught to medical physics residents. While professionalism education is required by CAMPEP during residency training, individual programs may have very different resources for and approaches to teaching this topic. Our goal is to collate the various approaches to teaching professionalism and to identify potential gaps in resources.

You are receiving this survey because you are listed as a residency program director. We would appreciate your cooperation in completing the survey, which should take no more than 20 mins. You may pause and return to the survey as needed. If you feel that another faculty is better suited to completing the survey, please forward it to them but each program should only submit one survey response.

Responses are anonymized and will be aggregated with the intention to publish the results.

Thank you!

What is your role in educating residents?	◯ Program Director<br>◯ Associate Program Director<br>◯ Other:
Describe your role:	__________________________
Describe your residency program's primary affiliation:	◯ Affiliated with a university<br>◯ Not affiliated with a university<br>◯ Other:
Describe your institution's affiliation:	__________________________
What area of medical physics does your residency program specialize in?	◯ Therapeutic<br>◯ Diagnostic<br>◯ Diagnostic + Nuclear Medicine
Enter the total number of residents currently in your program:	__________________________
How long is the training period at your program?	◯ 2 years<br>◯ More than 2 years
Does your institution have or is it affiliated with a medical physics graduate educational program?	◯ Yes<br>◯ No

10% Complete

.

There will be three sections of professionalism skills to be rated in separate tables: 1) Personal, 2) Inter-personal, and 3) Developmental.

Next to each personal skill listed, indicate whether:

You believe it is essential/desirable/non-essential for residents to develop this skill during training. You believe it is a teachable skill. Your program is currently teaching this skill. Personal Skills Essential? Teachable? Currently Teaching?

Emotional Self-Awareness: Recognizing how our emotions affect our performance. ______

______ ______

Accurate Self-Assessment: Knowing one's own inner resources, abilities and limits. __________________

Self-Confidence: A strong sense of one's self worth and capabilities. __________________

Transparency:
Maintaining integrity, acting congruently with one's values. __________________

Adaptability:
Flexibility in handling change. __________________

Achievement:
Striving to improve or meeting a standard of excellence. __________________

Initiative:
Readiness to act on opportunities. __________________

Optimism:
Persistence in pursuing goals despite obstacles and setbacks. __________________

35% Complete

.

Next to each interpersonal skill listed, indicate whether:

You believe it is essential/desirable/non-essential for residents to develop this skill during training. You believe it is a teachable skill. Your program is currently teaching this skill. Interpersonal Skills Essential? Teachable? Currently Teaching?

Empathy:
Sensing others' feelings and perspectives, and taking an active interest in their concerns. __________________

Organizational Awareness: Reading a group's emotional currents and power relationships. __________________

Service Orientation:
Anticipating, recognizing, and meeting customers' or clients' needs. __________________

Inspirational Leadership:
Inspiring and guiding individuals and groups. __________________

Change Catalyst:
Initiating or managing change. __________________

Influence:
Having impact on others. __________________

Conflict Management: Negotiating and resolving conflict. __________________

Teamwork and collaboration: Working with others towards a shared goal. Creating group synergy in pursuing collective goals. __________________

60% Complete

.

Next to each developmental skill listed, indicate whether:

You believe it is essential/desirable/non-essential for residents to develop this skill during training. You believe it is a teachable skill. Your program is currently teaching this skill. Developmental Skills Essential? Teachable? Currently Teaching?

Responsible behavior:
General professional behavior, including having composure and work ethic __________________

Ethical behavior:
Ethical behavior in professional, scientific, educational activities __________________

Respect for diversity behavior: Treating people equitably by understanding and appreciating perspectives, behaviors, and needs of people from all backgrounds ____________ ______

Delegation skills: Ability to prioritize tasks and transfer responsibilities to others with proper direction and authority ______ ______ ______

Time management skills: Coordinating tasks and activities to maximize the effectiveness of an individual's effort ______ ______ ______

Communication skills:
Includes all communication forms (e.g., written, oral, presentation, and marketing skills) _________________

Problem-solving skills:
Includes analytical and problem solving skills __________________

Professional vitality:
Includes well-being, resilience, work-life balance, burnout __________________

In this section, you will be asked about how professionalism is taught to physics residents.

85% Complete

.

Do you teach professionalism to physics residents?

◯ Yes
◯ No

How confident do you feel to teach professionalism skills to physics residents?

◯ Confident
◯ Neutral
◯ Not confident

Do you use the AAPM/RSNA/ASTRO/ACR/ARR/ARS Online Modules on Ethics and Professionalism to teach professionalism to physics residents?

◯ Yes
◯ No
◯ I'm not familiar with these modules

Do you feel that the AAPM/RSNA/ASTRO/ACR/ARR/ARS Online Modules on Ethics and Professionalism are sufficient for teaching professionalism to physics residents?

◯ Yes
◯ No
◯ I'm not familiar with these modules

Have you developed independent content for teaching professionalism to physics residents?

◯ Yes ◯ No ◯ In progress

Briefly describe the professionalism educational content that your program has developed.

____

Select all applicable methods your program uses to teach professionalism skills to physics residents:

☐ Observation of clinical staff
☐ Independent study and/or self-reflection by resident
☐ Retrospective discussion in response to event
☐ Prospective discussion
☐ Journal Club
☐ Didactic Lectures
☐ Online modules
☐ Case-based discussion
☐ Practice of simulated scenarios with actors
☐ Role play
☐ Other

Please describe the Other teaching methods used at your institution:

____

For the selected methods above, how often do you provide explicit training on professionalism skills to physics residents?

◯ None
◯ At least monthly
◯ At least quarterly
◯ At least annually
◯ Other:

Please describe the frequency with which you provide training on the professional skills listed above:

____

Are you satisfied with the effectiveness of the current professionalism training being provided to your physics residents?

◯ Satisfied
◯ Seeking continuous improvement
◯ Not satisfied

Are you assessing the professionalism skills of your residents?	◯ No, not assessing at all<br>◯ Yes, using a structured assessment method<br>◯ Yes, in an ad-hoc fashion<br>◯ Only when problems/issues arise
What methods are used to assess a physics resident's professionalism skills? (Select all that apply)	☐ Direct observation by rotation supervisor(s) or equivalent<br>☐ Indirect observation reported by others<br>☐ Professionalism-specific questions during an exam<br>☐ Other:
Please describe what methods you are using to assess a physics resident's professionalism skills:	__________________________________
How often do you assess a physics resident's professionalism skills?	◯ At least monthly<br>◯ At least quarterly<br>◯ At least annually<br>◯ Other:
Please describe how often you assess a physics resident's professionalism skills:	__________________________________
What methods are used to provide feedback to the physics residents regarding their professional skills during the course of your program? (Select all that apply)	☐ Verbal discussion with resident<br>☐ Written feedback with a score<br>☐ Written feedback with comments only<br>☐ Other: provide details or enter 'None' if applicable
Please describe the methods you are using to provide feedback to the physics residents regarding their professional skills during the course of your program:	__________________________________
Do you face barriers in implementing/expanding a formal curriculum for professionalism skills?	◯ Yes<br>◯ No
Select all the barriers that you face in implementing/expanding a formal curriculum for professionalism skills.	☐ Lack of time<br>☐ Lack of resources<br>☐ Lack of faculty expertise<br>☐ Lack of interest<br>☐ Other:
Please describe the barriers that you face in implementing/expanding a formal curriculum for professionalism skills:	__________________________________
What kind of resources do you wish to have to teach professionalism skills to residents (independent of the delivery format, e.g. in-person versus online)? (Select all that apply)	☐ Textbook/Reading Materials<br>☐ Didactic Lecture<br>☐ Self-reflection prompts<br>☐ Case studies<br>☐ Journal Club<br>☐ Simulated activities (i.e. role play, script and rubric) with clinical staff<br>☐ Simulated activities with professional actors<br>☐ Other:
Please describe the kind of resources you wish to have to teach professionalism skills to residents:	__________________________________

Have you received formal professionalism skills training (i.e. didactic course, workshop, etc.)?	◯ Yes<br>◯ No
How important is it for program staff to receive formal training in order to effectively teach professionalism skills to residents?	◯ Important<br>◯ Neutral<br>◯ Not important
Please share if there is anything else you wish to add about professionalism training:	__________________________________
