# Supplementary material for: Professionalism skills education in medical physics residency: Current state and perceived importance
Source: J Appl Clin Med Phys. 2025 Apr 24;26(6):e70096. doi: 10.1002/acm2.70096 (PMC12148774; doi:10.1002/acm2.70096)

1. There was 90% agreement amongst experts regarding relevance of questions, as compared with 85% agreement regarding clarity of questions.
2. In terms of CVI, the experts agreed that 98% of the questions were relevant and 97% of the questions were clear.
3. The CVI was calculated for each question by counting the number of experts rating the item as 3 or 4 and dividing that number by the total number of experts.
4. Questions and response options for which expert ratings did not agree, received a CVI <1.0, or which received suggestions for improvements were then either removed from the survey or refined accordingly.

Validation results for relevance ratings are displayed in the table below:

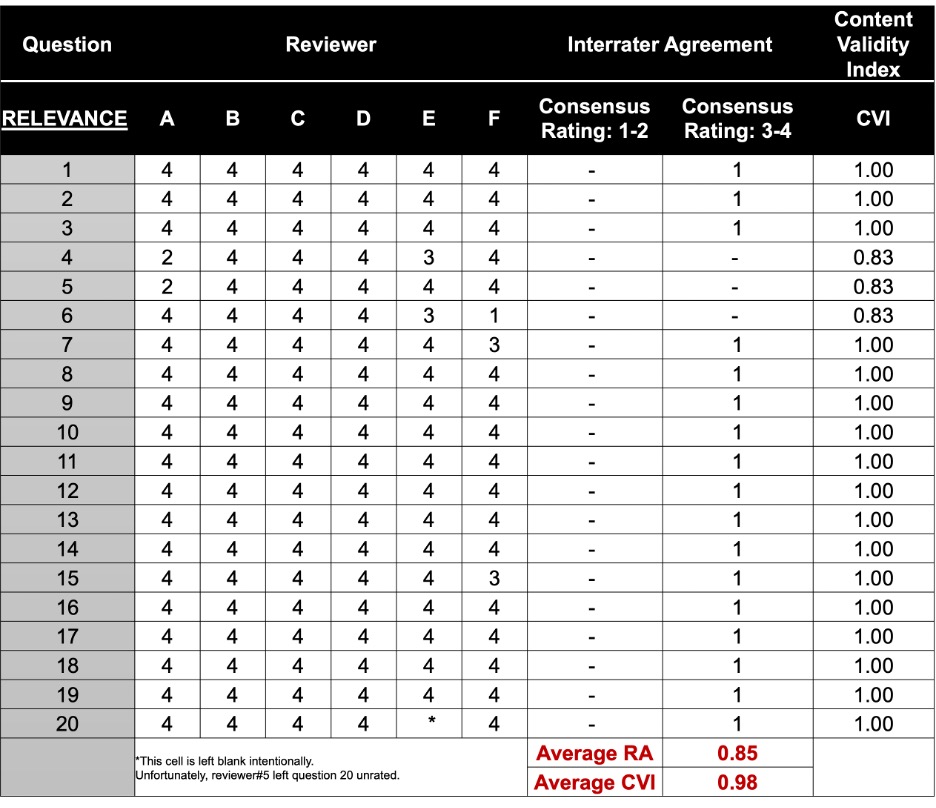

Supplement: Supplementary file 2 — Supporting Information [file ACM2-26-e70096-s001.docx]
